# Case Report: Expressive Speech Disorder in a Family as a Hallmark of 7q31 Deletion Involving the *FOXP2* Gene

**DOI:** 10.3389/fped.2021.664548

**Published:** 2021-08-20

**Authors:** Orsolya Nagy, Judit Kárteszi, Beatrix Elmont, Anikó Ujfalusi

**Affiliations:** ^1^Division of Clinical Genetics, Department of Laboratory Medicine, Faculty of Medicine, University of Debrecen, Debrecen, Hungary; ^2^Hospital of Zala County, Zalaegerszeg, Hungary; ^3^Department of Pediatrics, Hospital of Zala County, Zalaegerszeg, Hungary

**Keywords:** 7q31 deletion, *FOXP2*, expressive speech disorder, MLPA, case report

## Abstract

Pathogenic variants of *FOXP2* gene were identified first as a monogenic cause of childhood apraxia of speech (CAS), a complex disease that is associated with an impairment of the precision and consistency of movements underlying speech, due to deficits in speech motor planning and programming. *FOXP2* variants are heterogenous; single nucleotide variants and small insertions/deletions, intragenic and large-scale deletions, as well as disruptions by structural chromosomal aberrations and uniparental disomy of chromosome 7 are the most common types of mutations. *FOXP2*-related speech and language disorders can be classified as “*FOXP2*-only,” wherein intragenic mutations result in haploinsufficiency of the *FOXP2* gene, or “*FOXP2*-plus” generated by structural genomic variants (i.e., translocation, microdeletion, etc.) and having more likely developmental and behavioral disturbances adjacent to speech and language impairment. The additional phenotypes are usually related to the disruption/deletion of multiple genes neighboring *FOXP2* in the affected chromosomal region. We report the clinical and genetic findings in a family with four affected individuals having expressive speech impairment as the dominant symptom and additional mild dysmorphic features in three. A 7.87 Mb interstitial deletion of the 7q31.1q31.31 region was revealed by whole genome diagnostic microarray analysis in the proband. The *FOXP2* gene deletion was confirmed by multiplex ligation-dependent probe amplification (MLPA), and all family members were screened by this targeted method. The *FOXP2* deletion was detected in the mother and two siblings of the proband using MLPA. Higher resolution microarray was performed in all the affected individuals to refine the extent and breakpoints of the 7q31 deletion and to exclude other pathogenic copy number variants. To the best of our knowledge, there are only two family-studies reported to date with interstitial 7q31 deletion and showing the core phenotype of *FOXP2* haploinsufficiency. Our study may contribute to a better understanding of the behavioral phenotype of *FOXP2* disruptions and aid in the identification of such patients. We illustrate the importance of a targeted MLPA analysis suitable for the detection of *FOXP2* deletion in selected cases with a specific phenotype of expressive speech disorder. The “phenotype first” and targeted diagnostic strategy can improve the diagnostic yield of speech disorders in the routine clinical practice.

## Introduction

In the general population, childhood speech disorders are common clinical conditions affecting 1 in 20 preschool children (1). In regard to its possible causes, hearing impairment, autism, intellectual or psychomotor developmental disorders, and genetic syndromes are all in contention (2). Although the starting point and quality of speech in the first years of life may vary greatly, psychomotor alteration with or without the other clinical signs are indications for the child-neurologists to consult a clinical geneticist. Speech delay is one of the most frequent reason for genetic workup in early childhood. The lack of etiological diagnosis causes difficulty in giving proper genotype-phenotype correlations, in providing counseling with respect to possible outcome and treatment opportunities, and furthermore, in cases with a genetic background assessing the risk of recurrence. The genetic background of abnormal speech development is very heterogenous, therefore, phenotypical sub-characterization comes in handy for the clinical geneticist (3, 4). The type of the speech delay (global, expressive, receptive) and the presence of accompanying phenotypic signs and organ developmental disorders can orient the clinical geneticist. It is common that routine brain MRI scans, as part of the diagnostic workup, give negative results in childhood speech disorders, suggesting that brain abnormalities may be present at the sub-macroscopic level (3, 5). As such, the genetic workup is determined by the presence of the additional clinical symptoms of the patient. In case of a syndromic form, the characteristic symptoms of the assumed syndrome define the diagnostic methodology (e.g., Fragile-X syndrome–*FMR1* gene mutation analysis). In the majority of patients, the speech delay is not syndromic or the phenotype of the patient is not specific, as such, the currently available genome wide molecular (cyto)genetic techniques are chosen first. Chromosomal microarray is used to detect chromosomal copy number variations (CNVs), while whole exome sequencing (WES) is the main approach to identify mutations in the protein-coding genes.

Childhood apraxia of speech (CAS) is an uncommon motor speech disorder, defined as a higher-order motor system deficit of motor planning and programming of speech (3). The patients usually have an impaired speech development from infancy manifested as poor feeding, lack of babbling, delayed onset of first words, and limited number of spoken words. According to the American Speech-Language-Hearing Association consensus statement, there are three core diagnostic symptoms of CAS: (i) inconsistent errors on consonants and vowels; (ii) lengthened and disrupted coarticulatory transitions between sound and syllables; and (iii) inappropriate prosody (https://www.asha.org/policy/PS2007-00277). Differentiation from the other types of speech disorders such as articulation or phonological disorders, dysarthria, and stuttering is crucial for providing prognostic information to patients (3). This is a lifelong speech disorder. Many children with CAS also have language problems and literacy impairments that can influence their educational and employment outcomes (6).

*FOXP2* (Forkhead box protein P2) (MIM # 605317) was the first gene to be associated predominantly with speech and language disorders (7). It was recognized in a multigenerational “KE” family, wherein the affected members carried a point mutation at a highly conserved position (R553H) within the forkhead DNA binding domain of the protein. The *FOXP2* gene encodes a conserved transcription factor that is important in the development and functioning of the motor cortex, striatum, and cerebellum responsible for fine motor control (2, 8). Depending on the underlying genetic mechanism of *FOXP2* insufficiency, the individuals with *FOXP2*-related speech and language disorders can be categorized as “*FOXP2*-only” and “*FOXP2*-plus.” Patients with inactivating intragenic mutations in the *FOXP2* gene have only a speech and language disorder, therefore, these cases are classified as “*FOXP2*-only.” In “*FOXP2*-plus” individuals, the genetic background can be large copy number variants (i.e., contiguous gene deletions) (52% of affected individuals), structural variants (i.e., chromosome translocation or inversion) (8% of patients), or maternal uniparental disomy of chromosome 7 (UPD7) involving the *FOXP2* gene (11% of patients) (9). Microdeletions of different chromosomal regions (7q31, 2p15p16.1, 12p13.33, 16p11.2, 17q21.31) and other gene mutations (i.e., *GRIN2A, SETBP1*) have also been reported in CAS (7, 10–15).

The 7q31 microdeletion syndrome is an ultra-rare chromosomal anomaly (<1:1,000,000) characterized by a speech and language disorder. Individuals with larger deletions in this region have also been reported to display intellectual disability, dysmorphic features, developmental delay, and autism. About 52% of the *FOXP2*-related speech and language disorders are caused by 7q31q33 deletions that encompass the *FOXP2* gene and flanking DNA. In total, 80% of these deletions are *de novo* and the remaining cases are inherited in an autosomal dominant manner (9).

We report herein the clinical and genetic characterization of a large family with four affected individuals having speech impairment with variable severity and mild dysmorphic features, where the patients carry 7q31.1q31.31 deletion involving the *FOXP2* gene.

## Case Report

**Figure 1:**
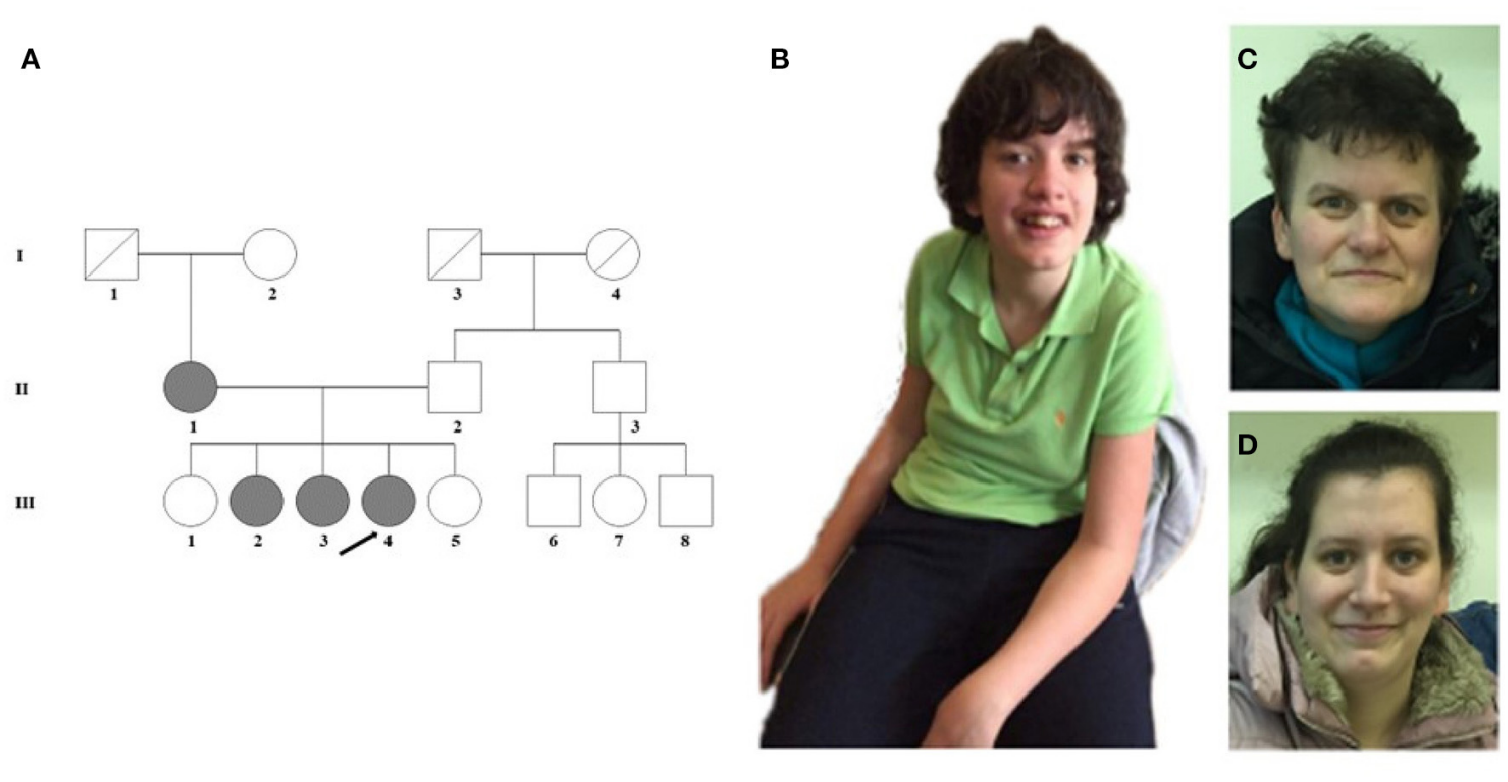
**(A)** Pedigree of the family. **(B)** The proband (III-4) shows scoliosis and mild facial dysmorphic features such as low forehead, slight hypertelorism, prominent nose, and wide lips. **(C)** The minor facial anomalies of the mother (II-1): maxillary hypoplasia, prominent nose, and pointed chin. **(D)** Face of the sister (III-3) with almost no dysmorphic features.

The female proband (III-4) was examined first from her family. She was born after an uneventful pregnancy at 38th weeks of gestation with a birth weight of 3,000 g from non-consanguineous parents (Figure 1A). Perinatal anamnesis was negative. At 10 months of age, a complicated febrile convulsion was observed and antiepileptic treatment was received for a while. She remained symptom free after finishing the therapy. Brain MRI was negative. No feeding difficulty was observed. Early motor development in infancy was almost normal. She walked independently at 15 months of age. However, the parents noticed that the overall movement was somewhat slow and sluggish, and she fell quite a lot of times. The neurologist detected muscle hypotonia and imbalance, and as a result, she received physiotherapy from the age of 2 years. Later, she got physiotherapy for scoliosis. She never reached harmonic walking at all and she walked with bent knees. Her speech development was delayed and it was retracted even further. She participated in special education considering her speaking disability that was mainly a motor speech impairment. Additionally, her receptiveness was also much below average. Hearing impairment was not detected. She was diagnosed to have a moderately severe psychomotor retardation based on the regular follow-ups of the pedagogical services that regularly check and analyse the development of children globally in different fields of mental and motor development. A child neurologist and a special needs teacher together took parts in every evaluation process performed regularly in this case. Table 1 summarizes the retracted development of the patient till school age. In spite of the profound delay, she presented for initial genetic evaluation at the age of 16 years. The delay was due to the attitude of the family who lived in a small village, where the mother and two sisters of the patient also suffered from speech impairment. At presentation, physical examination of the proband revealed normal body anthropometric data but several minor anomalies were described as follows: low forehead, slight hypertelorism, prominent nose, wide lips, long and slender fingers, and long toes (Figure 1B). Internal organs and external genitalia were normal. Neurological examination found very slow movements, thoracolumbar scoliosis, slight muscular atrophy with sluggish reflexes, convergent strabismus, and intellectual disability, but no abnormal reflexes or ataxia. The patient received regular and specific educational treatment by trained experts for speech and mental impairments with limited results.

**Table 1:** Clinical features of the affected family members and detailed developmental evaluation of the proband.

	**II-1 (Mother)**	**III-2 (Sister)**	**III-3 (Sister)**	**III-4 (Proband)**
Age (years)	49	27	25	16
Type of speech disorder	CAS	CAS	Dyspraxia of speech	CAS
Psychomotor development	Normal	Normal	Normal (IQ: 81)	Moderately severe psychomotor retardation (IQ: 31)
Minor anomalies	+	+	-	+
Education	Normal + speech therapy	Normal + speech therapy	Normal + speech therapy	Special school
Regular work	+	–	+	–
Sociability	Normal	Some difficulties	Normal	Severe delay
**Developmental evaluation of the Proband (III-4)**				
**Age of evaluation (year; month)**	Walking^*^	Hand manipulation^*^	Speech^*^	Sociability^*^
2; 11	19 months (broad base)	10 months (grasps objects for a few minutes)	6 months (gestures and voices)	22 months (hardly accepts unfamiliar people)
5; 3	22 months (some imbalance)	15 months (plays upon being instructed but not spontaneously)	9 months (some syllables)	12 months (cooperates for only a few minutes with the therapist)
7; 4	24 months (disharmonic)	18 months (plays with blocks)	9 months (slight improvement in understanding, says some words)	12 months (activity in cooperation is very simple)
17; 0	Wide based gait	Plays a lot with blocks, constructs complicated objects	Understands, says some words and simple sentences	Friendly, open for cooperation with the therapist

* *Age in months is equivalent to the indicated developmental age*.

*CAS, childhood apraxia of speech; IQ, intelligence quotient*.

The genetic examination of this patient was commenced with peripheral blood cytogenetics using the standard procedure, where the G-banded chromosome analysis showed a normal female karyotype (46,XX). Based on her speech delay and mild dysmorphic features, targeted FISH analysis was done to confirm or exclude the DiGeorge/Velo-cardio-facial syndrome using a locus specific FISH probe (DiGeorge/VCFS TUPLE1) (Cytocell, Rainbow Scientific Inc., Windsor, CT). The result of this test was normal. Array CGH was performed as the next routine diagnostic step using oligonucleotide microarray composed of ~60,000 probes, distributed through all the genome (*qChip Post*) (Quantitative Genomic Medicine Laboratories, S.L., Barcelona, Espana). Microarray analysis identified a pathogenic interstitial deletion on the long arm of chromosome 7 from 7q31.1 to 7q31.31 cytoband with the following coordinates: arr[GRCh37] 7q31.1q31.31(109745411_117482692) × 1. It was an ~7.73 Mb deletion, that alters the dosage of multiple reference genes, including the morbid gene *FOXP2*.

Detailed phenotype analysis of the family revealed speech difficulties among family members (Table 1; Figure 1A). The mother (II-1) of the proband had speech impairment since her childhood. She attended a normal primary school and worked in a factory as a manual laborer. She has some distinctive minor anomalies with maxillary hypoplasia, prominent nose, and pointed chin (Figure 1C). The older sister (III-2) of the proband, aged 27 years, has severe articulation problems and difficulties in social interactions and communication. She has abilities necessary for daily living, but she does not work. She is aware of her limitations and thus inhibited with unfamiliar people. The other affected sister (III-3) of the proband, aged 25 years, has a borderline IQ (81) with nasal speech and almost no dysmorphic features (Figure 1D). She has regular work and no communication difficulties besides the speech dyspraxia. The unaffected, completely healthy sisters (III-1 and III-5) and father (II-2) of the proband have an average intellectual status. No other family members are known to have any intellectual disability. It was obvious that our proband had the most severe clinical phenotype in the family; nonetheless, analysis of the other family members seemed reasonable.

Based on the array CGH result of the proband, targeted MLPA (Multiplex Ligation-dependent Probe Amplification) analysis was applied as a first-tier test on all family members to examine the deletion status of the *FOXP2* gene. For this analysis, SALSA MLPA Probemix P475-A1 FOXP1-FOXP2 (MRC-Holland, Amsterdam, The Netherlands) that contains probes for all exons of the *FOXP1* and *FOXP2* genes was used. This analysis identified the deletion of all exons of the *FOXP2* gene and no alteration in the *FOXP1* gene in the affected family members (II-1, III-2, III-3, III-4). The clinically healthy sisters (III-1, III-5) and the father (II-2) had normal MLPA results. According to the family history, the grandparents (I-1, I-2) did not have speech and language disturbances, therefore, we assumed that the 7q31 deletion was a *de novo* event in the mother. The grandparents were not available for genetic testing.

**Figure 2:**
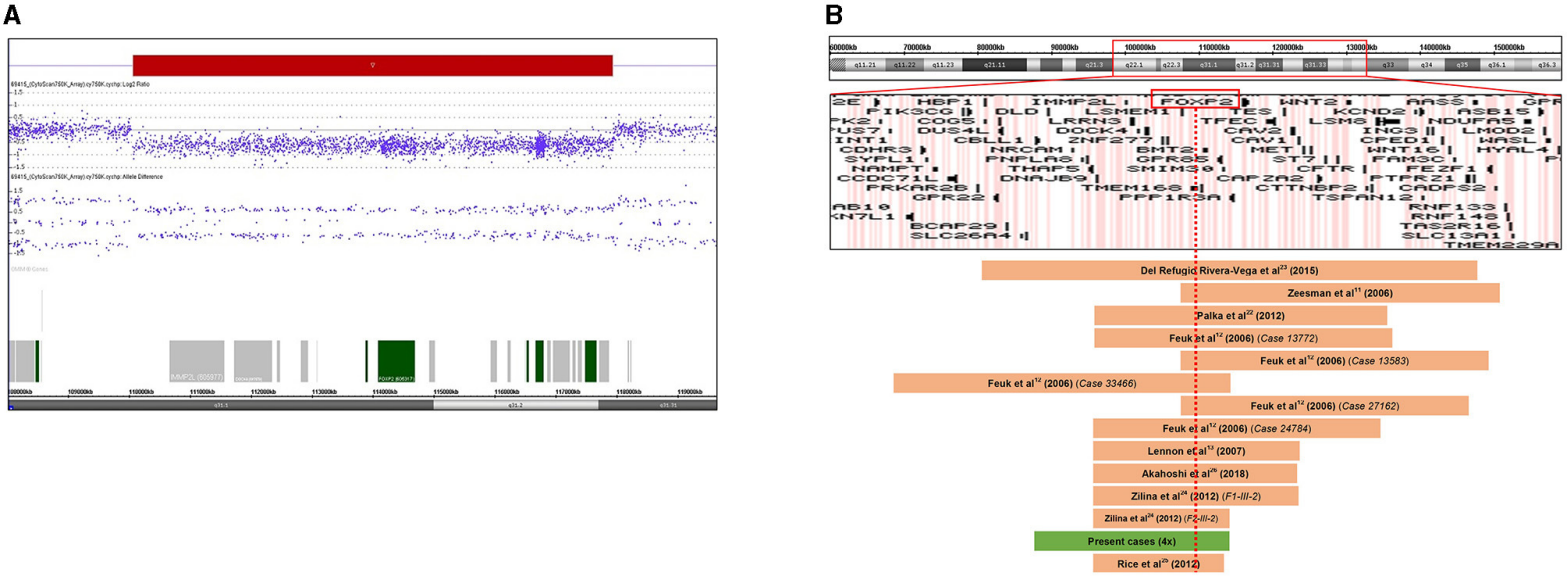
**(A)** Result of chromosome microarray analysis. A 7.87 Mb deletion at chromosome 7q31.1q31.31 (109708675-117578862). Signal intensity is plotted on a log2 scale, the deleted segment is shown as a red bar. Allele differences confirm the deletion. **(B)** Schematic representation of the 7q22q32 chromosomal region with gene content based on the UCSC Genome Browser. Deleted regions reported in previous cases are represented as brown bars, and the deletion of our patients is depicted as a green bar. *FOXP2* gene is highlighted by a red rectangle. The red dotted line shows that all reported deleted cases involve the *FOXP2* gene. Genomic positions refer to build19.

After all the aforementioned genetic investigations, we performed array CGH for all the affected family members using a higher resolution CytoScan 750K Array (Affymetrix, Thermo Fisher Scientific, Waltham, MA) to refine the extent of the 7q31 deletion, allowing an accurate determination of the breakpoints. In addition, we wanted to check the presence of concomitant CNVs that can explain the clinical heterogeneity of the affected family members. The higher resolution microarray revealed a heterozygous 7.87 Mb deletion with the following breakpoints: arr[GRCh37] 7q31.1q31.31(109708675_117578862) × 1 (Figure 2A). The size of the deletion and the breakpoints were exactly the same in every patient. The extent of the 7q31 deletion was slightly bigger than the one detected by the diagnostic microarray without altering the gene content (40 RefSeq genes, among them 20 OMIM morbid genes). No other concomitant pathogenic CNV was detected in the affected individuals. Figure 2B shows the genome map of the 7q22-7q32 region depicting the previously reported deleted cases and result of this study.

The observed microdeletion in the family has a great impact on family planning concerning the two fertile sisters with the CAS as they have a 50% chance to transmit the microdeletion to their children with an uncertain impact on speech development. The clinical geneticist has also emphasized that they are carriers of a *CFTR* gene deletion and, therefore, carrier testing of their partners is recommended. It was mentioned that targeted prenatal diagnostics is available in case of future pregnancies.

## Discussion

In this study, we present a large family with four affected individuals having an expressive speech impairment caused by the interstitial deletion of the 7q31 region involving the *FOXP2* gene. The 7q31 deletion was maternally inherited resulting in a distinct severity of speech disorder, mild behavioral alteration, and dysmorphic features in the affected family members.

Known genetic background of CAS is highly heterogeneous. Apart from the first-described *FOXP2* gene mutations, numerous other genes and chromosomal loci were discovered as causative factors in motor speech disorders. Highly penetrant variants usually affect common transcriptional pathways suggesting the essential role of transcriptional regulation in the normal speech development (4, 16). The *FOXP2* gene plays an important role in the speech and language development. Point mutations and deletions of *FOXP2* lead to verbal dyspraxia with impaired expressive and receptive language and are common in most individuals having CAS. Some patients may have a mild developmental delay as well (7). *FOXP2* is a member of the forkhead family of transcription factors, and homologs to other members of this family (*FOXP1, FOXP4*), having highly conserved domains (17). The gene is expressed in several structures in the brain including the cortical plate, basal ganglia, thalamus, inferior olives, and cerebellum where the FOXP2 protein may regulate the expression of other genes (18). The expression pattern is specific to subpopulations of neuronal cells in different structures (e.g., Purkinje cells in the cerebellum, deep layers of the cortex, and medium spiny neurons of the striatum). Disruptions of these cells during embryogenesis and postnatal development are risk factors for speech disorders. The human brain imaging studies indicate that *FOXP2* mutations alter the structure and function of the aforementioned brain structures (19, 20). The pathogenic variants of *FOXP2* are heterozygous and predicted to be loss-of-function changes, but the dominant negative effect of the mutant allele has also been suggested (2). Haploinsufficiency of the *FOXP2* gene results in impairments in the sequencing of movement and procedural learning leading to “Speech-language disorder 1” (*FOXP2*, MIM # 602081) in the affected individuals. In “*FOXP2*-only”-related disorders, non-verbal (performance) IQ is typically more preserved compared to verbal IQ. Core features of the disorder are childhood apraxia of speech and patients show difficulties in performing sequential orofacial movements, both linguistic and non-linguistic (9). They have an inability to generate syntactic grammar rules, impaired processing, and expressive language (4, 21).

The 7q31 deletion is a very rare chromosomal abnormality and familiar cases are even more unique. Zeesman et al. (11) first suggested that patients with chromosomal deletions involving 7q31 may define a new contiguous gene deletion syndrome characterized by developmental verbal dyspraxia. Speech and language deficits, articulation problems, and limited oral vocabulary are observed in all patients with haploinsufficiency of *FOXP2*. To date, <30 cases have been reported with interstitial 7q31 deletion encompassing the *FOXP2* gene. These cases usually carry different sized 7q31 deletion and consequently, they differ in clinical manifestation. Most of the 7q31 deletions reported are larger than 10 Mb and present a more complex clinical phenotype (22). The reported symptoms beside the speech impairment were the following: developmental delay, mild intellectual disability, and dysmorphic features (Table 2) (23–25). Intellectual disability, paranoid schizophrenia, and unilateral sensorineural hearing loss were described as CAS-associated symptoms only in single cases emphasizing their unknown genetic background (13, 26, 27). To the best of our knowledge, there are only two family studies with interstitial 7q31 deletion reported to date with one or two affected individuals and showing the core phenotype of *FOXP2* haploinsufficiency. Rice et al. (25) reported a detailed clinical assessment (speech, language, cognition, motor functions) of a moderately affected mother and her son with a severe apraxia of speech. Both of them carried a very small, 1.57 Mb deletion on chromosome 7q31 detected by array CGH. The deleted region involved only three genes: *FOXP2, MDFIC*, and *PPP1R3A*. Because the last two genes have not been associated with speech or language disorders, the clinical assessment of these patients provided informative phenotypic data on *FOXP2* haploinsufficiency. Their findings confirmed that *FOXP2* haploinsufficiency can disrupt development in cognition, speech, language, and sensorimotor domains. In the second family study, the authors described the clinical and molecular characterization of two familial cases with speech impairment, developmental delay, and congenital anomalies. They compared the phenotype of the affected patients with deletions of *FOXP2* inherited paternally and maternally (24). The authors did not find a significant difference due to the parental origin of the 7q31 deletion in the investigated two families. They could not confirm the hypothesis published earlier by Feuk et al. (12) that the loss of maternal *FOXP2* should be relatively benign while the loss of paternal *FOXP2* yields severe speech problems because of the differential parent-of-origin expression of the *FOXP2* locus. The clinical findings of our presented cases could not support this assumption either.

**Table 2:** Cytogenetic, molecular, and clinical data of patients with 7q deletion encompassing the *FOXP2* gene.

**References**	**Chromosomal region**	**Deletion size (Mb)**	**Speech and language**	**Other features**
Del Refugio Rivera-Vega et al. (23)	7q22.3q32.1	23.1	Language delay	Short stature, motor delay, craniofacial dysmorphism, microcephaly, hand anomalies, intellectual disability
Zeesman et al. (11)	7q31.2q32.2	16	Dyspraxia	Hypotonia, malformed ears, down-turned mouth, brachycephaly
Palka et al. (22)	7q31.1q31.3	14.8 (mosaic)	Dyspraxia	Mild psychomotor delay, high arched palate, lordosis
Feuk et al. (12)	7q31.1q31.3 (*Case 13772*)	15	Dyspraxia	Psychomotor delay, cognitive impairment, behavioral disturbances
	7q31.2q32.3 (*Case 13583*)	15	Dyspraxia	Psychomotor delay, cognitive impairment, ASD-like
	7q22q31.3 (*Case 33466*)	15	Dyspraxia	Psychomotor delay, cognitive impairment
	7q31.2q32 (*Case 27162*)	13	Dyspraxia	Psychomotor delay, cognitive impairment, ASD
	7q31.1q31.3 (*Case 24784*)	11	Dyspraxia	Autism, craniostenosis
Lennon et al. (13)	7q31.1q31.31	9.1	Dyspraxia	Ptosis, plagiocephaly, hypertelorism, bulbous nose, moderate psychomotor delay
Akahoshi et al. (26)	7q31.1q31.31	8	NA	Mild intellectual disability, minor anomalies, paranoid schizophrenia
Zilina et al. (24)	7q31.1q31.31 (*F1-III-2*)	8.3	Dyspraxia	Developmental delay, failure to thrive, dysmorphic phenotype, positive Graefe symptom, urinary tract anomalies, autistic features
	7q31.1q31.2 (*F2-III-2*)	6.5	Dyspraxia	Developmental delay, slightly dysmorphic phenotype, mild ataxia, occasional agressive behavioral
Present cases	7q31.1q31.31 (*III-4*)	7.87	CAS	Muscle hypotonia and imbalance, thoracolumbal scoliosis, moderately severe psychomotor retardation, minor anomalies
	7q31.1q31.31 (*III-3*)	7.87	Dyspraxia	Borderline IQ (81), no dysmorphic features
	7q31.1q31.31 (*III-2*)	7.87	CAS	Difficulties in social interactions and communication
	7q31.1q31.31 (*II-1*)	7.87	CAS	Minor anomalies with maxillary hypoplasia, prominent nose and pointed chin
Rice et al. (25)	7q31.1q31.2	1.57	CAS	No major congenital anomalies or dysmorphic features

*ASD, autism spectrum disorder; CAS, childhood apraxia of speech; FOXP2, forkhead box protein P2; IQ, intelligence quotient; Mb, megabase; NA, not applicable*.

It is noteworthy that the 7.87 Mb deletion detected in our proband (III-4) by array CGH covers the two-thirds of the 7q31 region that is known as the autism susceptibility locus 9 (AUTS9, MIM # 611015) as well. A meta-analysis of genome studies on autism or autism spectrum disorders (ASD) found a significant linkage to 7q31 suggesting that this chromosomal region is likely to harbor a susceptibility gene for autism. Although there are contradictory findings on the direct correlation between *FOXP2* variants and ASD, new data emphasize the misregulation of the target genes controlled by *FOXP2* in the downstream signaling pathways as the possible explanation of the autistic features of some of the *FOXP2* mutated patients (28). Autistic features were not observed in the affected members in the presented family, although the oldest patient (III-2) shows a more severe speech impairment and limited communication with unfamiliar people.

The clinical heterogeneity among the affected individuals in this family remains to be elucidated. According to literature data, the mechanism of the phenotypic manifestation of the CNVs and their incomplete penetrance remain largely unclear (29). Recently, it has been reported that differentially methylated regions inside CNVs may be one of the mechanisms of incomplete penetrance of inherited CNVs associated with neurodevelopmental disorders (30). Vasilyev et al. reported differential DNA methylation of intragenic CpG sites of the *IMMP2L* gene located in a critical region for the autism susceptibility locus on chromosome 7q (AUTS1). The authors suggest a partial compensation of *IMMP2L* gene haploinsufficiency in healthy CNV carriers by reducing the DNA methylation level (31).

The rearrangements of the *FOXP2* gene are considered rare events, probably because of the limitations of the targeted investigation used. Our results contribute to the better understanding of the behavioral phenotype of *FOXP2* disruptions and can aid in the identification of patients. We emphasize the importance of the careful evaluation of speech and language disturbances, focusing on the discrepancy between verbal and non-verbal abilities, lack of behavioral problems, hyperactivity, and autistic features that are frequently associated with speech delay. Our results also emphasize the importance of a targeted MLPA analysis suitable for the detection of *FOXP2* deletion and can improve the diagnostic yield of speech impairment in routine practice. Early molecular diagnosis is highly beneficial for patients as it can help in the assessment of the possible outcome and risk of recurrence.

## Conclusion

To the best of our knowledge, this is the first report of a family with four affected individuals carrying 7q31 deletion involving the *FOXP2* gene and presenting phenotypic variability both in speech impairment and in other symptoms. The maternally inherited *FOXP2* deletion provides additional support to the previously described role of *FOXP2* haploinsufficiency as a causative factor in speech disorder.

## Ethics Statement

The studies involving human participants were reviewed and approved by GINOP Ethics Committee. The patients/participants provided their written informed consent to participate in this study.

## Author Contributions

ON performed the MLPA and the high-resolution microarray analysis and wrote the first draft of the manuscript. JK collected the clinical data and contributed to writing of the manuscript. BE contributed to clinical data collection and phenotypic description. AU performed the supervision and edited the writing. All authors contributed to the article and approved the submitted version.

## Conflict of Interest

The authors declare that the research was conducted in the absence of any commercial or financial relationships that could be construed as a potential conflict of interest.

## Publisher's Note

All claims expressed in this article are solely those of the authors and do not necessarily represent those of their affiliated organizations, or those of the publisher, the editors and the reviewers. Any product that may be evaluated in this article, or claim that may be made by its manufacturer, is not guaranteed or endorsed by the publisher.
